# Cell type-specific alterations in fatty acid metabolism in neuronal subpopulations of schizophrenia and construction of a diagnostic model

**DOI:** 10.3389/fpsyt.2026.1770038

**Published:** 2026-04-21

**Authors:** Cui Zhao, Liang Zhang, Ping Yang, Liang Li, Weiqi Zeng, Weiqi Xie

**Affiliations:** 1 School of Clinical Medicine, Hunan Brain Hospital, Hunan University of Chinese Medicine, Changsha, Hunan, China; 2 Provincial Key Laboratory of Traditional Chinese Medicine (TCM) Diagnostics, Hunan University of Chinese Medicine, Changsha, Hunan, China

**Keywords:** schizophrenia, single-cell sequencing, fatty acid metabolism, machine learning, immune dysregulation

## Abstract

**Objective:**

This study integrates single-cell and bulk RNA-seq to investigate cell type-specific alterations in fatty acid metabolism-related genes in the dorsolateral prefrontal cortex (DLPFC) of schizophrenia (SCZ) patients and to evaluate their potential as diagnostic biomarkers.

**Methods:**

Integrating single-cell sequencing data (9 SCZ patients and 14 controls) and Bulk RNA-seq data (GSE174407, GSE107638), cell subpopulation identification and annotation were performed using Seurat. Key genes were identified by integrating differential gene screening, transcriptional regulatory network construction (SCENIC), pseudotime analysis (Monocle 2), and functional enrichment. A multi-gene diagnostic model was established using LASSO regression. Model performance was validated using ROC curves, nomograms, and immune cell correlation analysis. Finally, an MK-801-induced mouse SCZ model was used to validate the expression of key genes via qPCR.

**Results:**

The study found that specific neuronal cell subtypes (e.g., CUX2+ NeuN and OPRM1+ NeuN) were significantly upregulated in SCZ, and the differentially expressed genes (DEGs) in these cells (e.g., *HSP90AA1*, *HSPA1A*, *PTPRO*) were significantly enriched in fatty acid metabolism pathways. Further regression analysis identified five key genes associated with SCZ pathogenesis (*ACAA1*, *ACAT2*, *ACSS1*, *PSME1*, and *S100A10*). Subsequent analysis indicated that these genes not only participate in inflammatory responses in neuronal cells, showing significant negative correlations with inflammatory genes (*p* < 0.05), but are also closely related to disease diagnosis and prognosis. A diagnostic model and nomogram for SCZ were constructed based on these genes. The area under the ROC curve (AUC) for the model was 0.856 in the training cohort and 0.779 in the validation cohort, indicating reliable predictive performance for SCZ diagnosis. The SCZ risk predicted by the nomogram closely matched the actual risk. Furthermore, the Decision Curve Analysis (DCA) curve showed that the central gene curve was above the gray line, indicating a significant net benefit from using the nomogram to predict SCZ risk. Finally, significant differential expression of related genes was also found in SCZ mice (*p* < 0.001).

**Conclusion:**

This study reveals cell type-specific dysregulation of fatty acid metabolism in SCZ and provides a robust five-gene diagnostic model with translational potential.

## 1 Introduction

Schizophrenia (SCZ) is a complex psychiatric disorder with a global prevalence of approximately 1%. Its characteristic symptoms include positive symptoms (e.g., hallucinations, delusions), negative symptoms (e.g., affective flattening, social withdrawal), and cognitive impairment, imposing a heavy disease burden on patients and society (1). Although the etiology of SCZ is not fully elucidated, genetic factors and gene-environment interactions are considered core mechanisms in its pathogenesis (2). In recent years, breakthroughs in high-throughput sequencing technologies have revealed numerous genetic variants and molecular pathways associated with SCZ through genome-wide association studies (GWAS) and transcriptome analyses, such as synaptic function, immune regulation, and energy metabolism abnormalities (3–5). However, most of these studies are based on bulk tissue or mixed cell samples, making it difficult to resolve cell type-specific molecular mechanisms and limiting the development of precise diagnostic and therapeutic strategies (6).

Fatty acid metabolism, as a core component of energy supply, plays a dual role in the nervous system: on one hand, it provides substrates for neuronal activity, and on the other, its metabolites (e.g., arachidonic acid, docosahexaenoic acid) regulate neuroinflammation and synaptic plasticity (7). Multiple studies have shown that disturbances in fatty acid metabolism are closely associated with neuropsychiatric disorders such as Alzheimer’s disease and depression (8, 9). For instance, Mi et al. found that defective fatty acid degradation in astrocytic mitochondria in an Alzheimer’s disease model led to the accumulation of lipotoxic metabolites (e.g., long-chain acylcarnitines) and triggered neuroinflammatory responses by activating the TLR4/NF-κB signaling pathway in microglia, ultimately accelerating neuronal loss and cognitive decline (10). However, the role of fatty acid metabolism in SCZ remains controversial. Evidence from two research levels appears conflicting. On one hand, plasma metabolomics-based studies have observed significant abnormalities in long-chain fatty acid levels in SCZ patients, suggesting that perturbations in metabolic pathways may be directly associated with disease state (11). On the other hand, genome-wide association studies indicate that genetic polymorphisms in certain fatty acid metabolism-related genes are linked to the risk of developing SCZ (12). While both lines of evidence implicate fatty acid metabolism in SCZ, they reflect different biological dimensions: the former captures a peripheral metabolite phenotype under disease conditions, whereas the latter reveals an individual’s genetic predisposition. This inconsistency may stem from the inability of traditional bulk-tissue analyses to resolve cellular heterogeneity. Specifically, it remains unclear whether the observed metabolic disturbance represents a primary pathological change in specific neuronal subpopulations or merely a secondary reflection of systemic metabolic status. Thus, the apparent controversy underscores the need for cell-type-specific investigations.

The rise of single-cell sequencing technology provides a new perspective for revealing cellular heterogeneity in SCZ. For example, recent single-cell transcriptomic analyses have identified unique gene expression patterns in excitatory neuronal subpopulations (e.g., CUX2+ neurons) in the prefrontal cortex of SCZ patients, suggesting subtype-specific contributions to the disease (13). Furthermore, the integration of Bulk RNA-seq and single-cell data has been successfully applied to analyze metabolic pathways in Parkinson’s disease, revealing abnormalities in lipid metabolism pathways in oligodendrocytes (14). However, no study has yet systematically investigated the cell type-specific expression of fatty acid metabolism-related genes and their association with disease progression in SCZ.

To address this gap, this study integrates single-cell sequencing and Bulk RNA-seq technologies, focusing on neuronal subpopulations in the dorsolateral prefrontal cortex (DLPFC) of SCZ patients. It aims to systematically identify cell type-specific expression patterns and regulatory networks of fatty acid metabolism-related genes in SCZ patients; elucidate their relationship with disease progression and immune regulation; and based on these genes, construct a reliable SCZ diagnostic model to evaluate its clinical translational potential, thereby providing a theoretical basis for developing novel diagnostic biomarkers and targeted therapeutic strategies.

## 2 Materials and methods

### 2.1 Data sources

#### 2.1.1 Single-cell data

Single-cell RNA sequencing data were obtained from a publicly available dataset (PMID: 36223459), including dorsolateral prefrontal cortex (DLPFC) samples from 9 schizophrenia (SCZ) patients and 14 control subjects. The DLPFC was selected due to its established involvement in cognitive and executive dysfunctions associated with SCZ.

Single-cell transcriptomic data enable the characterization of gene expression patterns at the resolution of individual cells, allowing cellular heterogeneity to be resolved and cell type–specific molecular features to be examined. This approach is particularly suitable for investigating neuronal subpopulations in complex psychiatric disorders. The dataset provides sufficient sequencing depth and cell numbers to support reliable cell clustering, annotation, and downstream analyses.

#### 2.1.2 Bulk RNA-seq data

GSE174407 and GSE107638: Download unprocessed bulk transcriptome data of neuronal cells from the SCZ and control groups. GSE174407 was used as the training cohort for model development, while GSE107638 served as an independent external validation cohort to assess the model’s generalizability and robustness. Compared with single-cell data, bulk RNA-seq offers greater sample-level stability and is well suited for differential expression analysis and diagnostic model construction. The inclusion of independent bulk datasets allows external validation of findings and improves the robustness and generalizability of subsequent analyses.

### 2.2 Data preprocessing and quality control

Data were aligned to the human genome (GRCh38) using Cell Ranger V6.1.2. Single-cell data were processed using Seurat V4.1.1 (15), following general single-cell analysis quality control pipelines (16, 17). Cells with mitochondrial content >10%, hemoglobin content >5%, or expressing fewer than 200 or more than 50,000 genes were filtered out to remove low-quality cells. Data normalization, cell clustering, and dimensionality reduction were performed using the Seurat package (15, 17). The top 2000 highly variable genes were selected from the corrected expression matrix using the FindVariableFeatures function, followed by principal component analysis using the RunPCA function, retaining the top 20 principal components for further analysis.

### 2.3 Cell clustering and annotation

Batch effects were corrected using the RunHarmony function from the R package harmony (18). Cell clustering was performed using the FindClusters function (resolution = 0.9), and nonlinear dimensionality reduction was performed using the RunUMAP function. Cell populations were annotated based on marker genes (19).

### 2.4 Single-cell differential gene analysis and functional enrichment

Differentially expressed genes (DEGs) between different groups were analyzed using the FindMarkers function in the Seurat package. The screening criteria were set as *p* < 0.05 and an average fold change > 0.3. To account for multiple comparisons, we controlled the false discovery rate (FDR) using the Benjamini-Hochberg procedure. Statistical significance was set at FDR-corrected *p* < 0.1.

### 2.5 Gene set variation analysis

Using the GSVA package in R, the most enriched hallmark pathways (from the Molecular Signatures Database: https://www.gsea-msigdb.org/gsea/msigdb/index.jsp) in each cell from SCZ and Control samples were investigated. Then, the limma package was used to perform differential analysis of the enrichment scores of molecular pathways between the two groups of cells. |t value| > 4 and *p* < 0.05 were considered indicative of differentially enriched pathways between the two cell groups.

Definition of the Fatty Acid Metabolism Gene Set;The candidate gene pool for this study was derived from the Hallmark gene set HALLMARK_FATTY_ACID_METABOLISM in the Molecular Signatures Database (MSigDB), which comprises 185 genes (the complete list is provided in **Supplementary Table 1**). This gene set was used as the basis for identifying and filtering fatty acid metabolism-related genes in subsequent analyses.

### 2.6 Transcription factor analysis

To calculate the regulon specificity scores (RSS) for each cell subpopulation, we used the pySCENIC Python package for SCENIC analysis (20, 21). First, GRNBoost2 was used to infer co-expression modules between transcription factors (TFs) and candidate target genes. Next, RcisTarget was used to analyze genes in each co-expression module to identify enriched motifs (defining a regulon as a transcription factor and its potential direct target genes). Finally, AUCell was used to assess the activity of each regulon in each cell. This protocol followed the scalable pySCENIC workflow described by van de Sande et al (21).

### 2.7 Construction of protein-protein interaction network based on hub genes

The STRING database was used to search for interactions among hub genes to construct a network, which was then visualized using Cytoscape software.

### 2.8 Pseudotime analysis

Trajectory analysis was performed using the Monocle 2 package to reveal the differentiation trajectory of neuronal cells (22). The integrated expression matrix with batch effects removed was used as input data, and unit trajectories and evolutionary orders were inferred using default parameters. The graph_test function was used to identify highly variable genes associated with the cell trajectory (22).

### 2.9 Bulk RNA-seq differential gene analysis

Differential expression analysis between SCZ and control samples was performed using the limma (v3.56.2) package. Genes with a *p* < 0.05 were considered DEGs.

### 2.10 Univariate logistic regression analysis

Univariate logistic regression analysis was performed using the rms package in R to further screen the fatty acid metabolism-related DEGs in order to identify independent risk genes for disease pathogenesis. Genes with *p* < 0.05 were retained for further analysis.

### 2.11 Development of a diagnostic model based on fatty acid metabolism-related genes

The LASSO algorithm (23) from the lars package in R was used to further screen selected fatty acid metabolism-related DEGs. Subsequently, multivariate logistic regression analysis was performed using the rms package in R, and the optimal fatty acid metabolism-related gene signature was selected as diagnostic genes. A risk score (RS) was calculated based on the expression and coefficients of each diagnostic gene, and a diagnostic model was subsequently constructed.

### 2.12 Diagnostic model performance evaluation

The receiver operating characteristic (ROC) curve method from the pROC package was used to evaluate the effectiveness of the constructed diagnostic model in the training(GSE174407) and validation cohorts(GSE107638).

### 2.13 Construction and evaluation of the nomogram model

A diagnostic nomogram model was established using the rms package to predict the incidence of SCZ. Calibration curves and decision curve analysis (DCA) were used to evaluate the predictive ability and practicality of the model, respectively.

### 2.14 Correlation analysis between diagnostic genes and immune status

In the training cohort, the ssGSEA tool was used to evaluate scores related to immune features in the samples (24, 25) (**Supplementary Table 2**). In the analyzed datasets, there were no significant differences in demographic characteristics such as age, sex, or medication history between the case and control groups(*p* >0.05). Simultaneously, the cor function in R was used to calculate the correlation between significantly different diagnostic genes and immune cells in SCZ versus control samples.

### 2.15 Animal model validation

#### 2.15.1 Establishment of SCZ mouse model and behavioral testing

All animal experiments were approved by the Animal Ethics Committee of the University of Chinese Medicine (Approval No. SLBH-202103100001) and conducted in accordance with the institutional guidelines for the care and use of laboratory animals. The animal license number was SCXK (Xiang) 2019-0004.

Twenty 7-week-old male C57BL/6 mice (SPF, weight 19–20 g) were selected. Eight mice were randomly chosen as the control group, and the remaining eight mice were administered MK-801 powder (Sigma) at an injection dose of 0.6 mg/kg/d dissolved in saline, via intraperitoneal injection for 2 weeks to establish the SCZ model. The open field test was used to verify successful model establishment.

After behavioral testing, mice were humanely euthanized for tissue collection. Mice were first anesthetized by intraperitoneal injection of Tribromoethanol (Avertin) solution (Tigergene, REF: TG-Avertin-M) at a dose of 0.2 mL/10 g body weight (maximum 0.5 mL). The injection was performed on the right lower abdomen: the needle was inserted subcutaneously for 3–5 mm toward the head, then advanced at a 45° angle through the abdominal muscle into the peritoneal cavity. Upon confirmation of successful anesthesia (lack of pedal reflex), mice were immediately euthanized by decapitation using a laboratory animal decapitator (Zhenhua Biological Instruments, Cat#: Decapitator-630). The head was positioned in the guillotine slot, and the blade was rapidly depressed to sever the cervical spine. Residual tissue was cleared using surgical scissors (Jingulao, 20 cm straight sharp scissors) and forceps (BKMAM, 20 cm straight tip dressing forceps). Death was confirmed before proceeding with tissue collection.

#### 2.15.2 qPCR

To validate the expression changes of the five diagnostic genes (*ACAA1*, *ACAT2*, *ACSS1*, *PSME1*, and *S100A10*) in schizophrenia, we performed qPCR on prefrontal cortex tissue and peripheral blood samples from the MK-801-induced SCZ mouse model described above. After behavioral testing, mice were sacrificed, and prefrontal cortex (50 mg per sample) and whole blood (200 µL per sample) were rapidly collected from 8 control and 8 model mice.

For RNA extraction from prefrontal cortex tissue, 30–50 mg of tissue was placed into a 2 mL RNase-free grinding tube, cut into small pieces, and immediately frozen in liquid nitrogen for 2–3 seconds. Then, 1200 µL of TriQuick Reagent and grinding beads (one 4 mm steel bead + two 3 mm steel beads) were added, and the tissue was thoroughly homogenized. After standing at room temperature for 5 minutes, the homogenate was centrifuged at 12,000 g for 10 minutes at 4 °C, and the supernatant was transferred to a new tube. Chloroform (0.2 volumes) was added, vigorously shaken for 30 seconds, and incubated at room temperature for 2–3 minutes. Following centrifugation at 12,000 g for 10 minutes at 4 °C, the upper aqueous phase was collected. Isopropanol (0.5 volumes) was added, mixed by inversion, and precipitated at room temperature for 10 minutes. The RNA pellet was collected by centrifugation at 12,000 g for 10 minutes at 4 °C, washed with 75% ethanol, and centrifuged again. The pellet was air-dried and dissolved in DEPC-treated water, then stored at –80 °C.

For peripheral blood RNA extraction, total RNA was isolated from 200 µL of whole blood using the Tissue/Cell Total RNA Isolation Kit (Vazyme, RC113-01) according to the manufacturer’s protocol. Briefly, 1 mL of LB10 buffer was added to the blood, vortexed, and incubated at room temperature for 5 minutes. After adding 0.2 mL of RNA Extraction Agent, the mixture was vigorously shaken, incubated for 3 minutes, and centrifuged at 10,000 g for 15 minutes at 2–8 °C. The upper aqueous phase was transferred to a new tube, mixed with an equal volume of absolute ethanol, and transferred to an RNA Spin Column. The column was centrifuged at 12,000 g for 30 seconds, washed twice with WB10 buffer, and centrifuged again to remove residual ethanol. RNA was eluted with 100 µL of RNase-free water and stored at –80 °C.

Reverse transcription was performed using the HiScript 1st Strand cDNA Synthesis Kit (+gDNA wiper, Vazyme, R412-01). The 20 µL reaction system contained 1 µg total RNA, 5 µL of 4× HiScript IV RT SuperMix, 1 µL Oligo(dT)20VN, 2 µL Random Primers, and RNase-free ddH_2_O. The reaction was incubated at 37 °C for 15 minutes (simultaneous gDNA removal), followed by 85 °C for 5 seconds, and then held at 4 °C.

Quantitative real-time PCR was performed on an ABI 7500 Real-Time PCR System using the One Step RT-qPCR SYBR Green Kit (Vazyme, Q226-01) (26). Each 20 µL reaction contained 10 µL of 2× One Step SYBR Green Master Mix, 1 µL each of forward and reverse primers (10 µM), 2 µL cDNA, and 6 µL RNase-free ddH_2_O. The amplification program was 94 °C for 30 seconds, followed by 45 cycles of 94 °C for 5 seconds and 60 °C for 30 seconds, with a default melt curve analysis. β-actin was used as the internal control, and relative expression was calculated using the 2^-^ΔΔCt method (27). All primer sequences are listed in **Supplementary Table 3**.

## 3 Results

### 3.1 Genes significantly upregulated in CUX2+ NeuN and OPRM1+ NeuN neuronal subtypes in SCZ are enriched in fatty acid metabolism pathways

**Figure 1:**
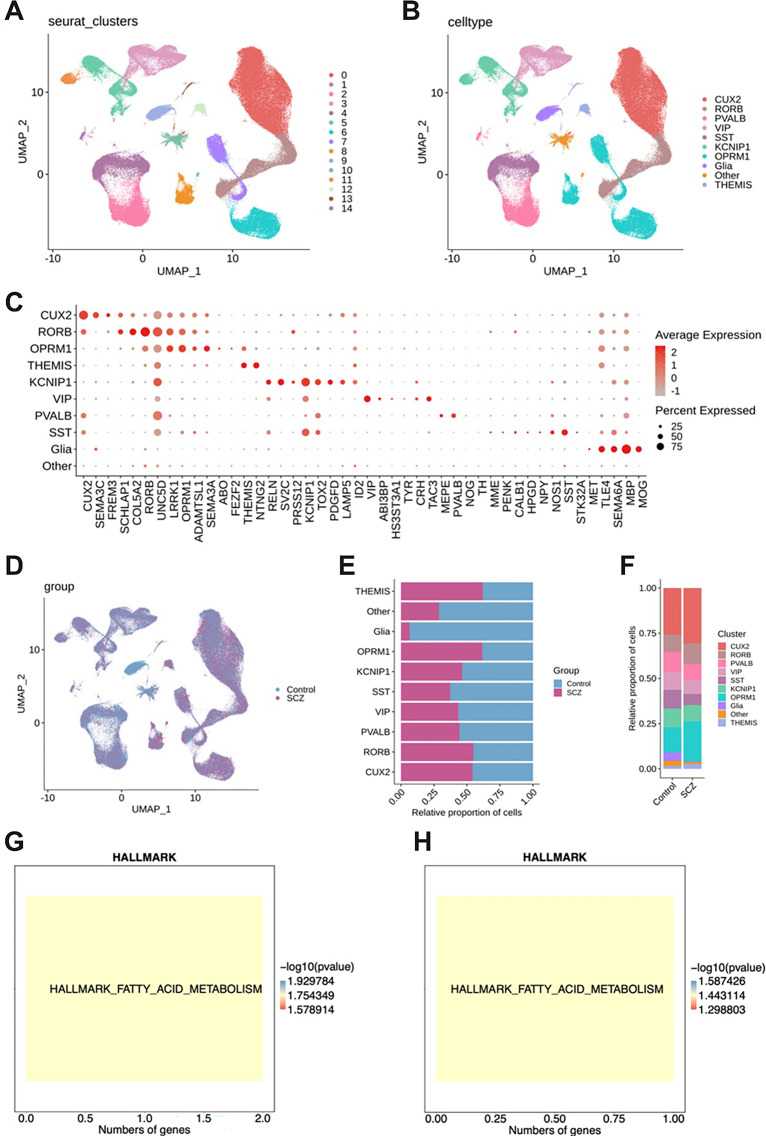
Single-cell annotation results. **(A)** Cell clustering results. **(B)** Cell annotation results. **(C)** Marker gene expression. **(D)** UMAP plot showing cell origins. **(E)** Proportion of various neuronal cell types in SCZ and Control groups. **(F)** Cell proportion in each sample. **(G)** Genes significantly upregulated in CUX2+ NeuN in SCZ are enriched in the fatty acid metabolism pathway. **(H)** Genes significantly upregulated in OPRM1+ NeuN in SCZ are enriched in the fatty acid metabolism pathway.

Clustering of 190,122 neuronal cells from 23 samples resulted in 10 annotated cell types based on marker genes: CUX2+ NeuN, RORB+ NeuN, PVALB+ NeuN, VIP+ NeuN, SST+ NeuN, KCNIP1+ NeuN, OPRM1+ NeuN, Glia, THEMIS+ NeuN, and other cells (**Figures 1A–C**). Analysis of the proportion of these cells in SCZ and Control groups showed that the proportions of CUX2+ NeuN and OPRM1+ NeuN were increased in SCZ samples (**Figures 1D–F**). **Figures 1G, H** shows that genes significantly upregulated in CUX2+ NeuN and OPRM1+ NeuN in SCZ were enriched in fatty acid metabolism pathways (**Figures 1G, H**; **Supplementary Figure 1**).

### 3.2 Identification of specific transcriptional regulatory programs in neuronal subtypes

**Figure 2:**
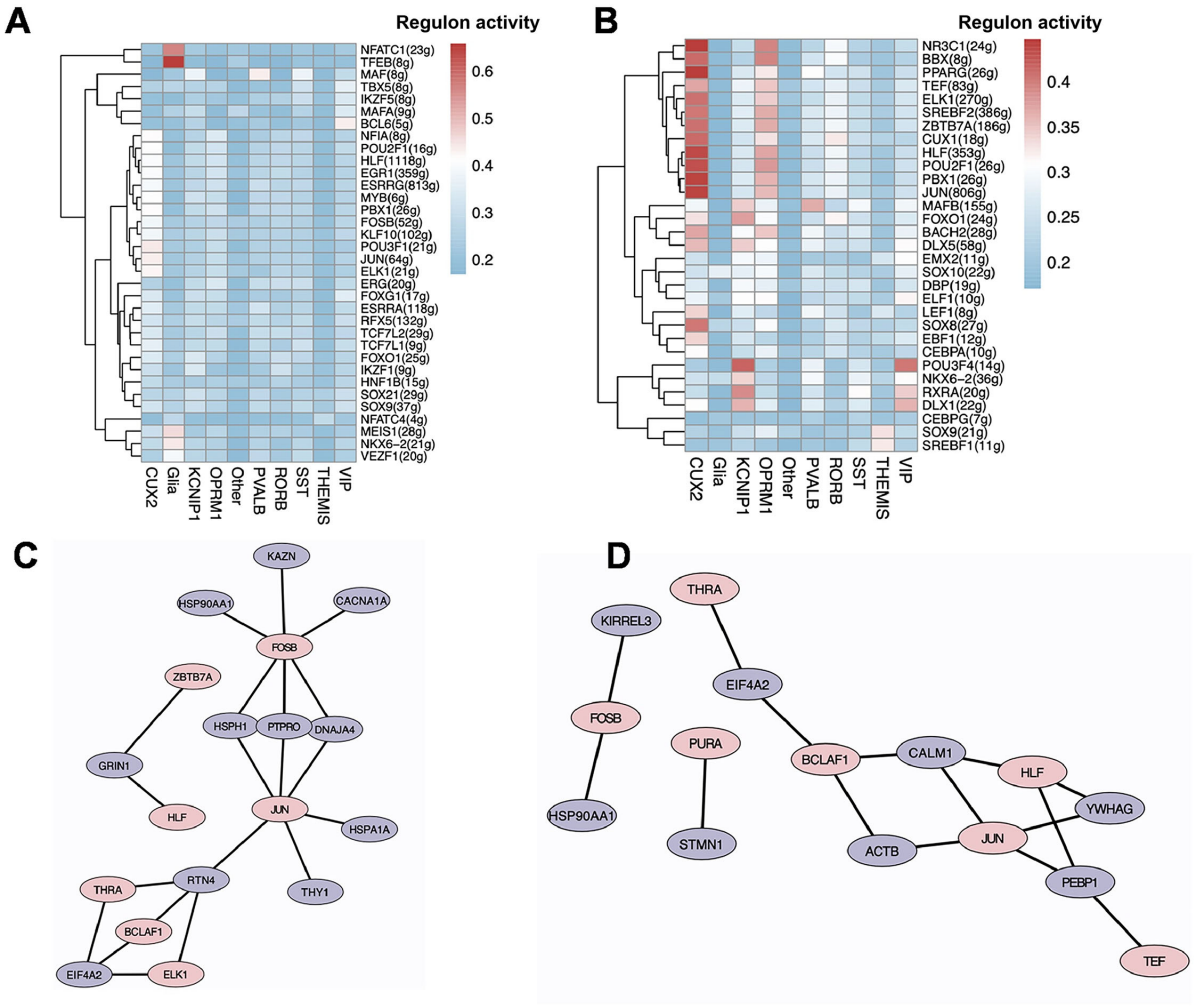
Transcriptional regulatory analysis of neuronal cell subtypes. **(A)** Heatmap showing the top 5 enriched transcription factors (TFs) in each cell subtype in the Control group. **(B)** Heatmap showing the top 5 enriched transcription factors (TFs) in each cell subtype in the SCZ group. **(C)** Network diagram showing TFs (pink) and their predicted target genes in CUX2+ NeuN. Purple nodes represent differentially expressed genes (DEGs) in CUX2+ NeuN. **(D)** Network diagram showing TFs (pink) and their predicted target genes in OPRM1+ NeuN. Purple nodes represent DEGs in OPRM1+ NeuN.

As mentioned, neuronal subtypes exhibit extensive transcriptomic heterogeneity, prompting us to examine regulon activity in these subtypes. By calculating the regulon specificity score (RSS), we observed that JUN and PPARG were specifically enriched in CUX2+ NeuN and OPRM1+ NeuN of SCZ samples (**Figures 2A, B**). Based on SCENIC results, we further constructed a transcriptional regulatory network to explore the transcriptional mechanisms of neuronal subtypes. The results indicated that JUN and FOSB were key TFs regulating the DEGs of the CUX2+ NeuN and OPRM1+ NeuN subtypes (**Figures 2C, D**). Among the 11 predicted target differentially expressed genes (DEGs) in CUX2+ NeuN, *HSP90AA1*, *HSPA1A*, and *PTPRO* are associated with fatty acid metabolism; among the 8 predicted target DEGs in OPRM1+ NeuN, *HSP90AA1* is associated with fatty acid metabolism.

### 3.3 Differentiation relationships among neuronal cell subtypes

**Figure 3:**
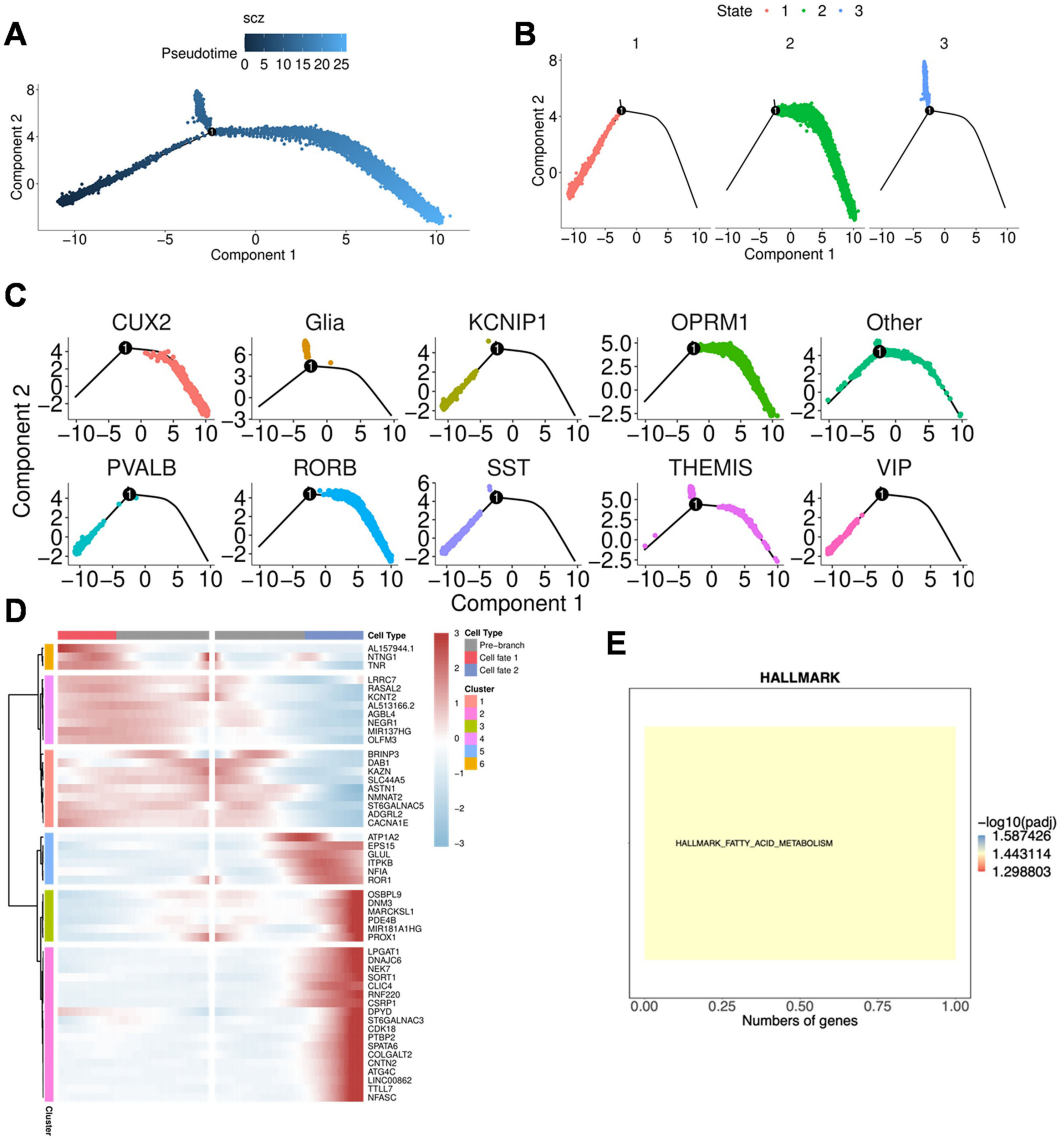
Trajectory analysis of neuronal cell subtypes. **(A–C)** Charts generated using Monocle software show the differentiation trajectory of neuronal cells, with colors representing different pseudotime states. **(A)** trajectory states. **(B)** The three fate states of neuronal cells: State 1 (pre-branch state), State 2, and State 3. **(C)** The cell states in which different subtypes are predominantly distributed. **(D)** Heatmap showing the expression patterns of 50 branch-dependent genes across the three states, identified using the Branch Expression Analysis Modeling (BEAM) method based on differentially expressed genes (DEGs) from each neuronal cell subtype. **(E)** Enrichment analysis results for branch gene cluster 5.

To understand the dynamic changes of neuronal cells, we constructed a cell trajectory to infer the differentiation relationships among neuronal subpopulations. Among these neuronal subtypes, we observed three distinct states (**Figures 3A,B**). The CUX2+ NeuN and OPRM1+ NeuN subtypes were predominantly distributed in State 2 (**Figure 3C**). Through Branch Expression Analysis Modeling (BEAM) analysis, we identified 50 branch-dependent genes that regulate the cell differentiation process from the pre-branch state (State 1) to Cell Fate 1 (State 2) and Cell Fate 2 (State 3) (**Figure 3D**). Classifying these 50 genes yielded 6 clusters of branch-related genes. We found that genes in cluster 5 were primarily enriched in fatty acid metabolism-related pathways, and their expression increased from the pre-branch state to Cell Fate 2 (**Figures 3D, E**).

### 3.4 Construction of a diagnostic model based on five fatty acid-related DEGs

**Figure 4:**
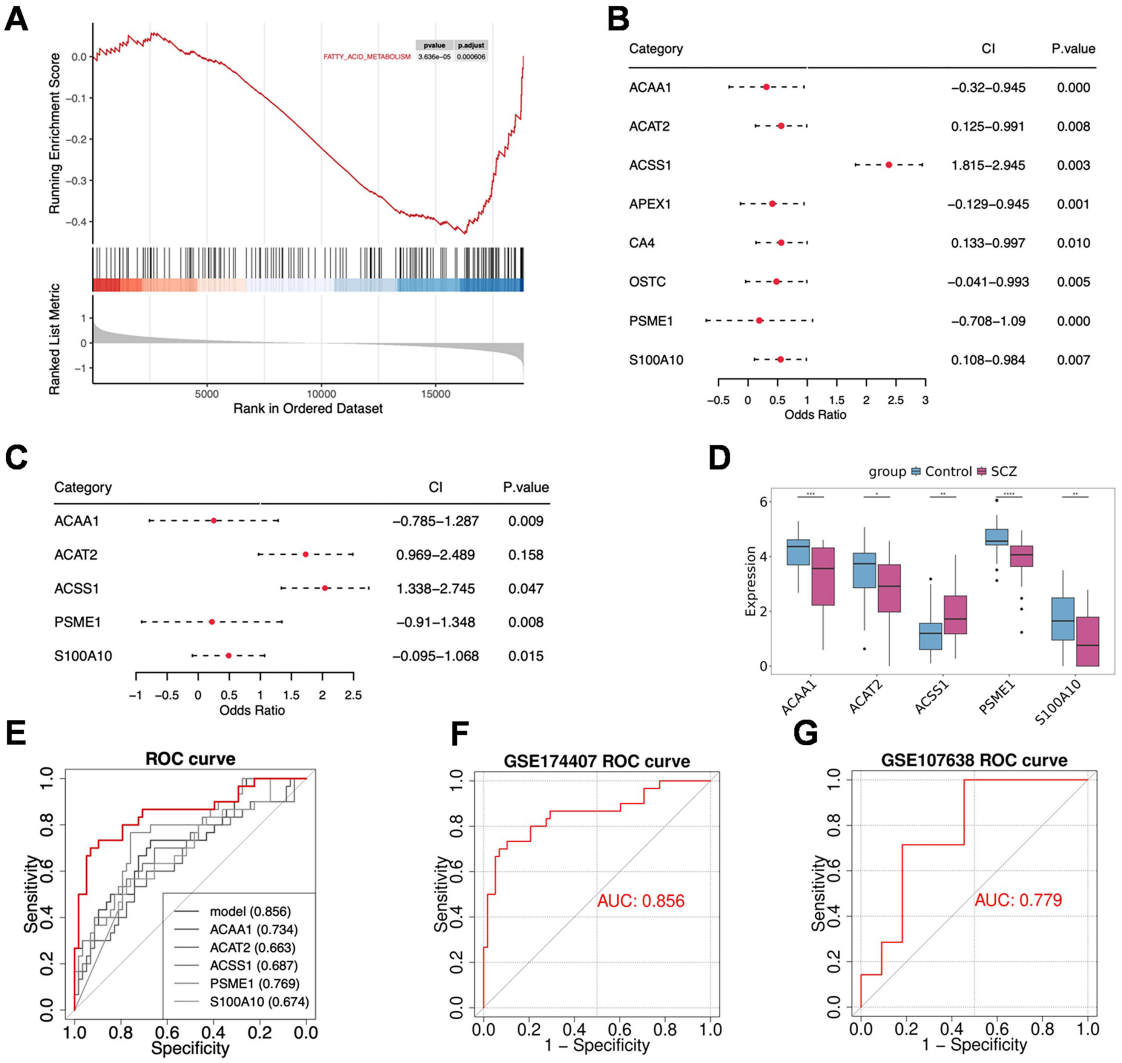
Establishment of the five-gene signature diagnostic model. **(A)** GSEA results show significant activation of the fatty acid metabolism pathway in SCZ samples. **(B)** Univariate logistic regression analysis confirms the association of fatty acid metabolism-related genes with SCZ occurrence. **(C)** Multivariate regression analysis of the five diagnostic biomarkers used for model construction. **(D)** Expression levels of the five genes in SCZ and Control groups, *p < 0.05, **p < 0.01, ***p < 0.001, ****p < 0.0001. **(E)** Receiver operating characteristic (ROC) curves of the model and single genes in the training cohort (GSE174407). **(F)** ROC curve of the model in the training cohort (GSE174407). **(G)** ROC curve of the model in the validation cohort (GSE107638).

To assess the involvement of fatty acid metabolism in SCZ pathogenesis, we first performed Gene Set Enrichment Analysis (GSEA) on the bulk RNA-seq data. The results revealed that the fatty acid metabolism pathway was significantly activated in SCZ samples compared to controls (**Figure 4A**), supporting the rationale for focusing on fatty acid metabolism-related genes in subsequent analyses.

Univariate logistic regression analysis was used to screen candidate genes for predicting SCZ onset from 158 fatty acid metabolism-related genes. These results indicated that 8 genes could be used as potential diagnostic biomarkers (*p* < 0.05, **Figure 4B**). Multivariate regression analysis identified five diagnostic biomarkers for model construction (**Figure 4C**): *ACAA1*, *ACAT2*, *ACSS1*, *PSME1*, and *S100A10*. Among these, *ACSS1* was significantly highly expressed in SCZ samples (*p* < 0.01, **Figure 4D**). We found that the predictive power of the multi-gene model was superior to that of any single gene (**Figure 4E**). Furthermore, we used the training cohort (GSE174407) and the validation cohort (GSE107638) to evaluate the predictive performance of the established model. The area under the ROC curve (AUC) for the model was 0.856 in the training cohort and 0.779 in the validation cohort, indicating reliable predictive performance for SCZ diagnosis (**Figures 4F, G**).

### 3.5 Construction of the diagnostic nomogram model

**Figure 5:**
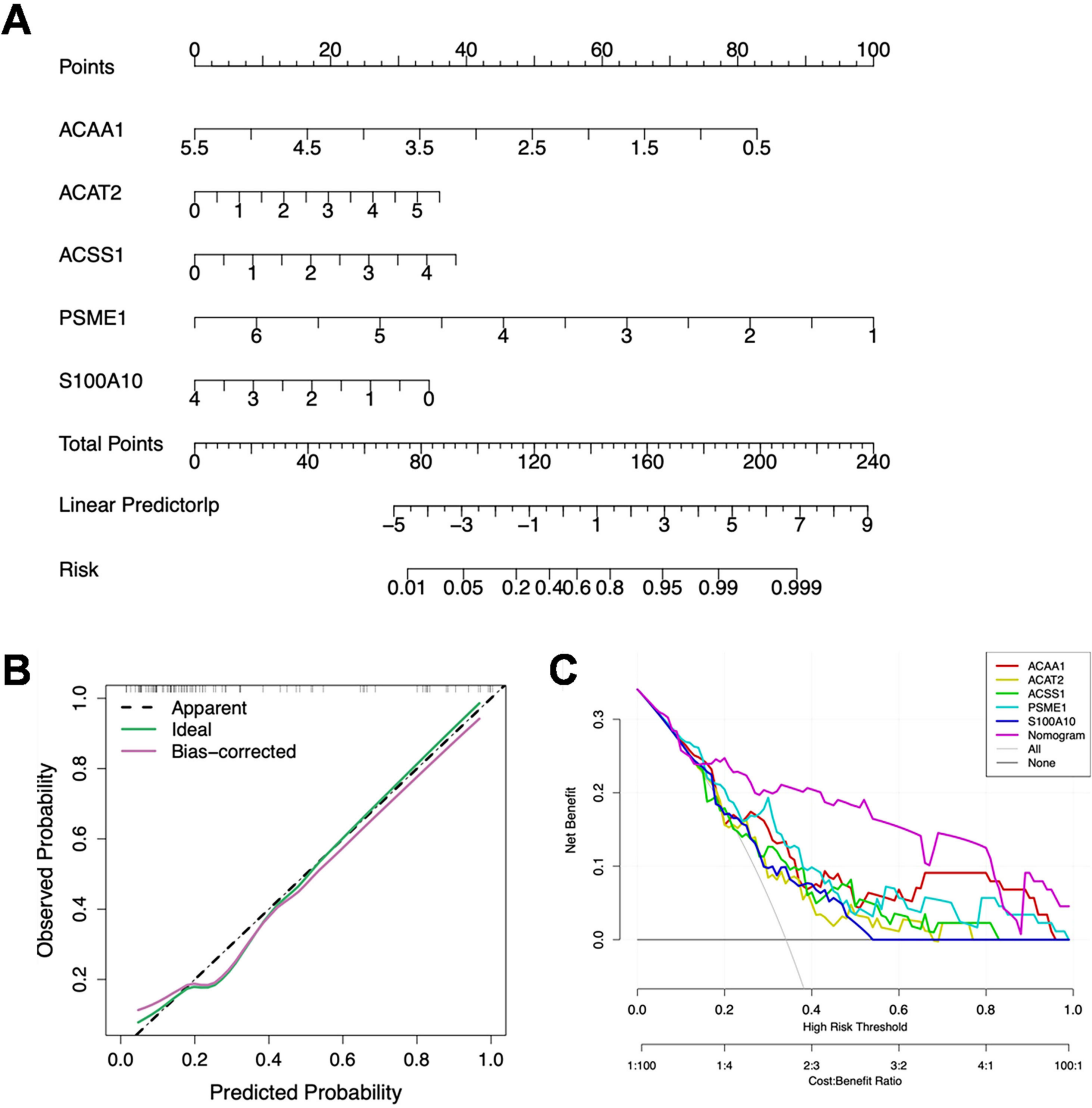
Construction of the nomogram model for SCZ diagnosis. **(A)** Nomogram based on the 5 genes for predicting SCZ risk. **(B)** Calibration curve for assessing the diagnostic potential of the model. **(C)** DCA curve for evaluating the practical efficacy of the model.

A nomogram model was generated based on the five genes to predict SCZ risk. As shown in **Figures 5A**, each predictor is projected upwards to a “Points” scale at the top of the nomogram, obtaining a score from 0 to 100. The total points of the five predictors are calculated to predict the probability of SCZ risk. The calibration curve showed that the predicted SCZ risk closely matched the actual risk (**Figure 5B**). Furthermore, the DCA curve showed that the central gene curve was above the gray line, indicating a significant net benefit from using the nomogram to predict SCZ risk (**Figure 5C**).

### 3.6 Correlation between fatty acid metabolism genes and inflammatory genes

**Figure 6:**
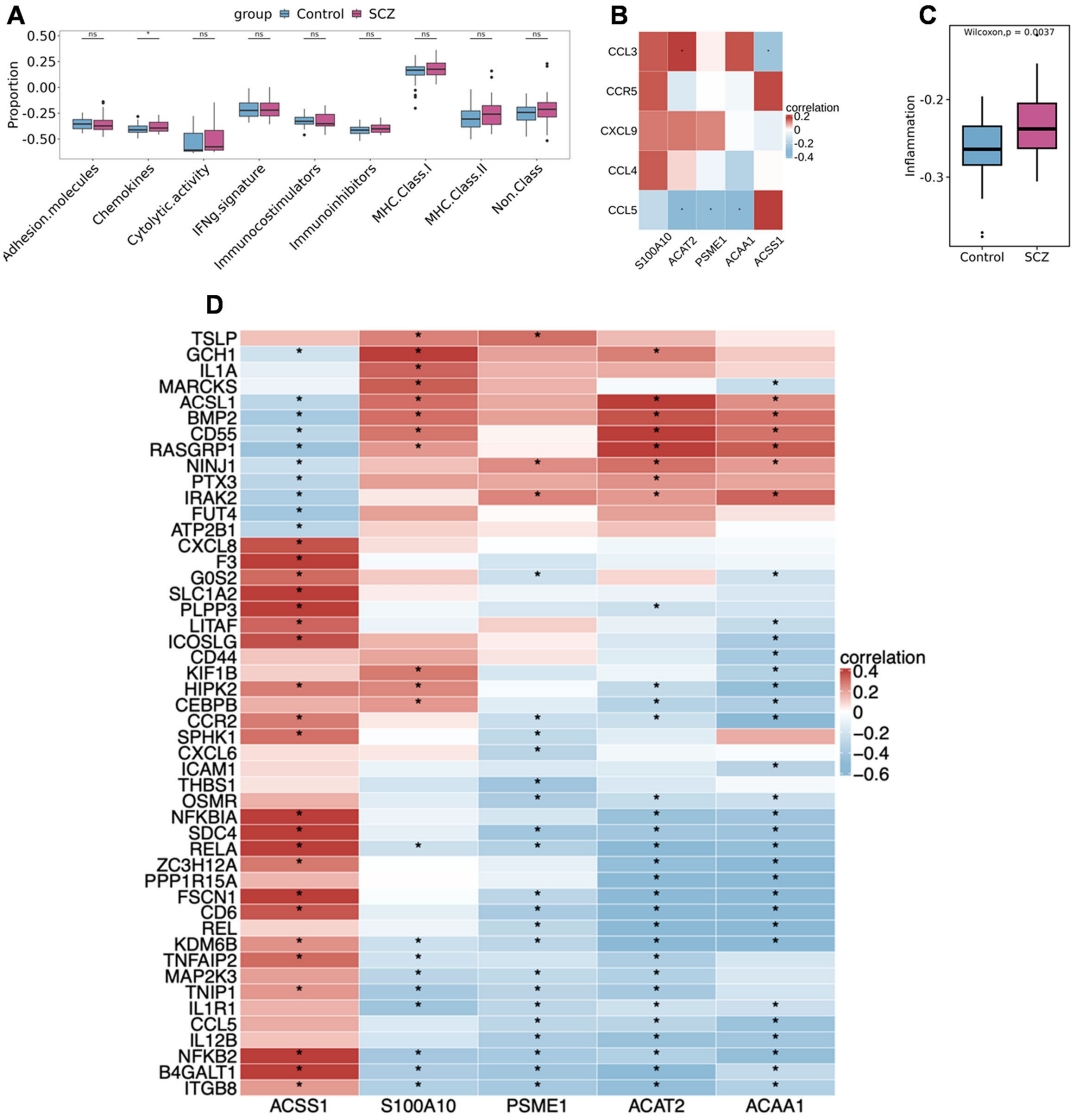
Analysis of immune feature scores in neuronal cells from the SCZ and Control groups in the training cohort. **(A)** Analysis of immune feature scores in neuronal cells from SCZ and Control groups in the training cohort. **(B)** Correlation between model genes and Chemokines-related genes (red indicates positive correlation, blue indicates negative correlation, *p < 0.05). **(C)** Inflammation response scores are significantly elevated in SCZ neuronal cells in the training cohort. **(D)** Correlation between model genes and Inflammation-related genes (red indicates positive correlation, blue indicates negative correlation, *p < 0.05).

**Figure 7:**
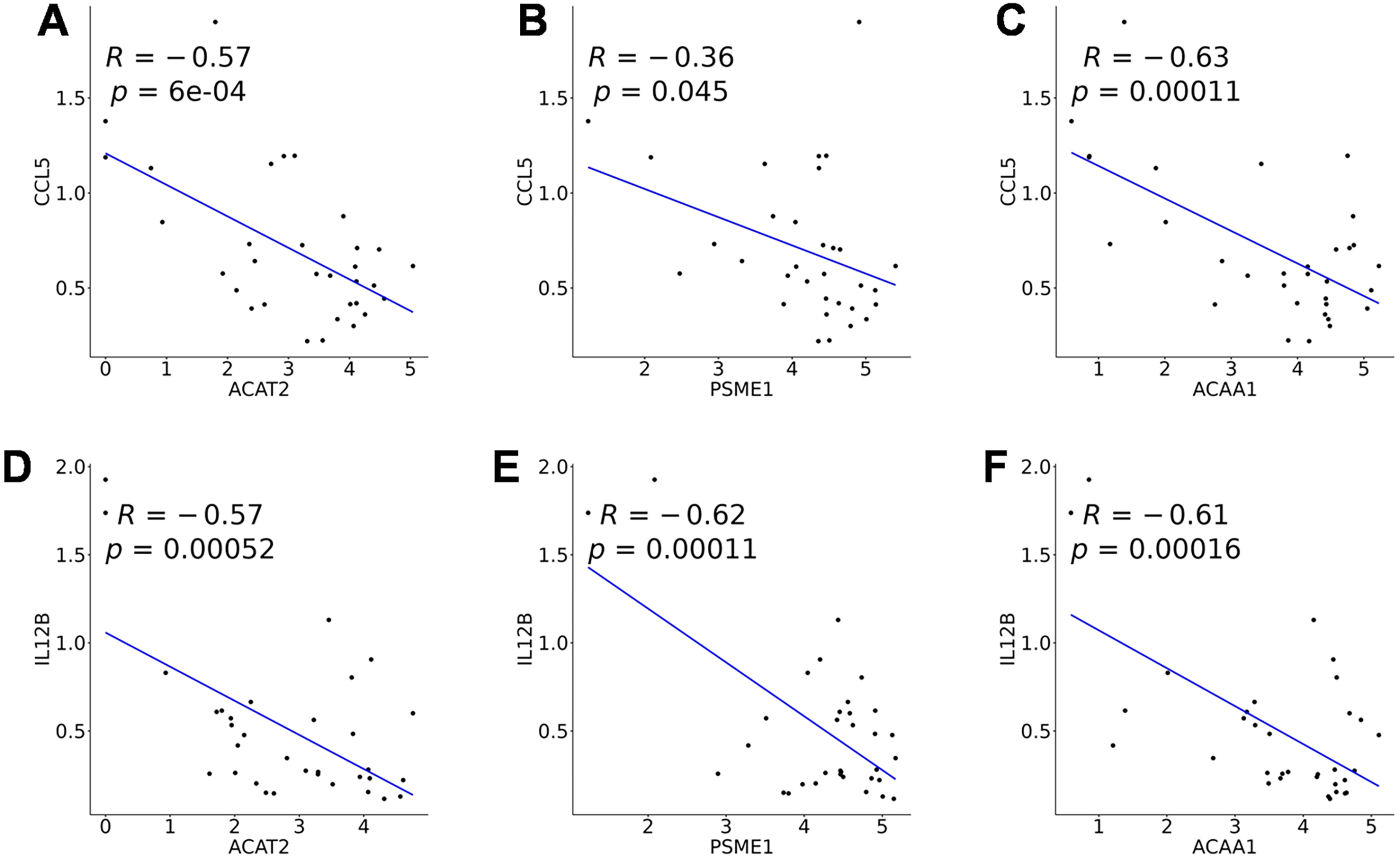
Correlation analysis between fatty acid metabolism genes and inflammation-related genes. **(A)** Correlation analysis between *ACAT2* gene and CCL5 gene. **(B)** Correlation analysis between *PSME1* gene and CCL5 gene. **(C)** *ACAA1* gene shows a significant negative correlation with CCL5 gene. **(D)** Correlation analysis between *ACAT2* gene and IL12B gene. **(E)** Correlation analysis between *PSME1* gene and IL12B gene. **(F)** Correlation analysis between *ACAA1* gene and IL12B gene.

Analysis of immune feature scores in neuronal cells from the training cohort revealed that the Chemokines score was significantly higher in SCZ neuronal cells compared to the Control group (**Figure 6A**). Analysis of the correlation between the model genes and Chemokines-related genes revealed that *ACAT2*, *PSME1*, and *ACAA1* showed significant negative correlations with the CCL5 gene (*p* < 0.05, **Figures 6B**, **7A–C**). Analysis of inflammation response scores in neuronal cells from the training cohort revealed that the Inflammation score was significantly higher in SCZ neuronal cells compared to the Control group (**Figure 6C**). *ACAT2*, *PSME1*, and *ACAA1* showed significant negative correlations with the IL12B gene (*p* < 0.05, **Figures 6D**, **7D–F**).

### 3.7 Validation of SCZ mouse model establishment

**Figure 8:**
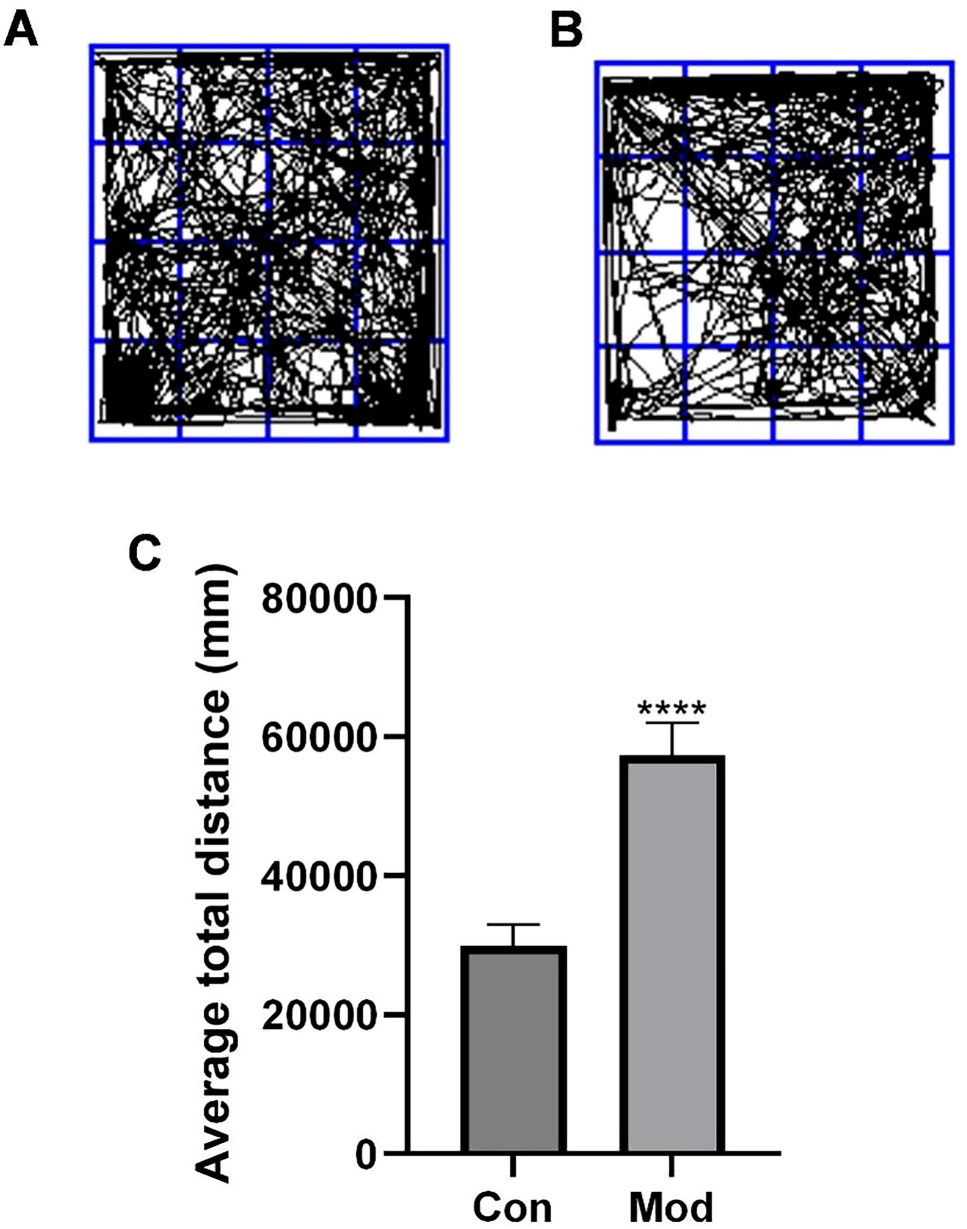
Total distance traveled by mice in the open field test. **(A)** Movement trajectory map of SCZ mice in the open field test. **(B)** Movement trajectory map of control mice in the open field test. **(C)** Total distance traveled by mice in the open field test. ****p < 0.0001.

To further validate our previous findings, we established an SCZ mouse model by intraperitoneal injection of the NMDA receptor antagonist MK-801. The behavioral phenotype of the model mice was assessed using the open field test. As shown in **Figures 8A, B** , compared to control mice, the model mice exhibited significantly increased movement trajectories in the open field, manifested as increased total movement distance crossing the central and peripheral zones. Quantitative analysis showed that the mean movement distance of the model group (57,352.60mm) was significantly higher than that of the control group (29,870.25 mm) (*p* < 0.0001, **Figure 8C**), indicating that MK-801 successfully induced psychomotor excitation in mice, mimicking the positive symptoms of SCZ patients, and confirming the successful establishment of the SCZ model.

### 3.8 Significant differential expression of five diagnostic genes in SCZ mice

By reviewing relevant literature, we found that *ACSS1*, *PSME1*, and *S100A10* are associated with psychiatric disorders such as depression and schizophrenia (4, 28, 29). although there is currently limited literature directly linking *ACAA1* and *ACAT2* to psychiatric disorders, their involvement in fatty acid metabolism pathways suggests potential relevance to SCZ pathology. After establishing the SCZ mouse model, we validated the expression of all five diagnostic genes in both dorsal prefrontal cortex and peripheral blood samples.

**Figure 9:**
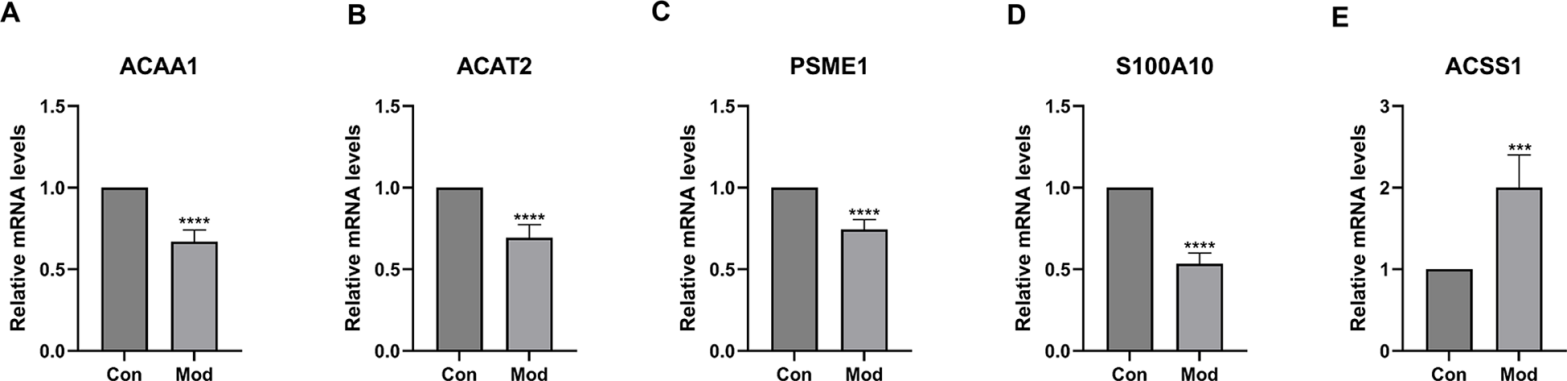
Gene expression in the dorsal prefrontal cortex of SCZ mice and control group. **(A)** *ACAA1* gene expression in control and SCZ groups. **(B)** *ACAT2* gene expression in control and SCZ groups. **(C)** *PSME1* gene expression in control and SCZ groups. **(D)** *S100A10* gene expression in control and SCZ groups. **(E)** *ACSS1* gene expression in control and SCZ groups. ***p < 0.001, ****p < 0.0001.

In prefrontal cortex tissue, the results showed that the expression of *ACAA1*, *ACAT2*, *PSME1*, and *S100A10* was significantly higher in the control group compared to the SCZ group (*p* < 0.0001, **Figures 9A–D**), while *ACSS1* expression was significantly higher in the SCZ group compared to the control group (*p* < 0.001, **Figure 9E**).

**Figure 10:**
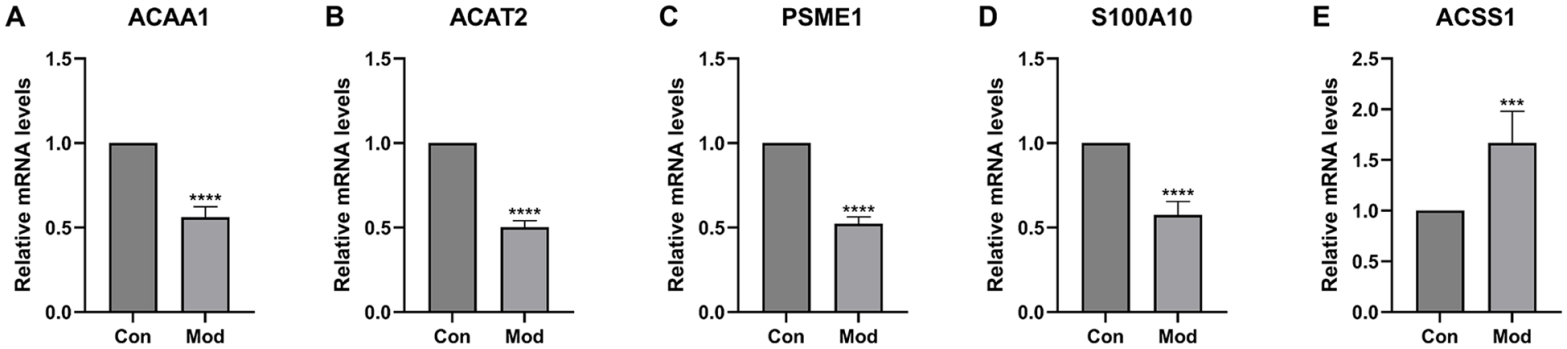
Gene expression in peripheral blood of SCZ mice and control group. **(A)** *ACAA1* gene expression in control and SCZ groups. **(B)** *ACAT2* gene expression in control and SCZ groups. **(C)** *PSME1* gene expression in control and SCZ groups. **(D)** *S100A10* gene expression in control and SCZ groups. **(E)** *ACSS1* gene expression in control and SCZ groups. ***p <0.001, ****p < 0.0001.

In peripheral blood samples, consistent expression patterns were observed: *ACAA1*, *ACAT2*, *PSME1*, and *S100A10* were significantly downregulated in the SCZ group (*p* < 0.0001, **Figures 10A–D**), whereas *ACSS1* was significantly upregulated (*p* < 0.001, **Figure 10E**), demonstrating that the transcriptional alterations of these fatty acid metabolism-related genes are not limited to brain tissue but can also be detected peripherally.

These findings are highly consistent with the data obtained from human dorsal prefrontal cortex and support the potential utility of these five genes as non-invasive diagnostic biomarkers.

## 4 Discussion

This study found that among tens of thousands of neurons from 23 samples, the proportions of two excitatory neuronal subtypes, CUX2+ NeuN and OPRM1+ NeuN, were significantly increased in the dorsolateral prefrontal cortex (DLPFC) of SCZ patients. Further functional enrichment analysis revealed that the significantly upregulated genes in these two cell populations were highly enriched in fatty acid metabolism pathways. This result suggests that abnormal fatty acid metabolism does not occur diffusely in all neurons but is confined to specific excitatory subtypes. This finding echoes the results of Skene et al. (6), who used GWAS data to predict that SCZ risk genes are preferentially enriched in upper-layer excitatory neurons, and complements the lack of cellular heterogeneity resolution in cross-tissue transcriptome-wide association studies based on bulk transcriptomes, such as those by Gusev et al. (13).

Next, using the SCENIC algorithm, we observed significantly enhanced regulatory activity of the transcription factors JUN and PPARG in the aforementioned two neuronal subtypes. PPARG, known to be involved in fatty acid β-oxidation, shows increased regulatory activity in our analysis, which might be associated with neuronal energy demands (30); Concurrently, JUN, an AP-1 family member linked to oxidative stress responses, also exhibits heightened activity (31). The observed co-activation of PPARG and JUN leads to the hypothesis that an interaction between metabolism and inflammation could be a contributing factor in SCZ pathology. Notably, PPARG has been reported to be involved in modulating microglial inflammatory responses (32). Our findings raise the possibility that its dysregulation might be associated with both neuronal metabolic states and local immune environment, though this requires further investigation.

Pseudotime analysis further illustrated this temporal relationship. Monocle 2 divided neuronal differentiation into three states, with fatty acid metabolism-related genes significantly upregulated in State 2 (dominated by CUX2+ and OPRM1+ subtypes), which coincided with the peak of inflammatory pathway activation. Among the 50 branch-dependent genes identified by the BEAM method, cluster 5 was not only enriched in fatty acid metabolism but also overlapped with synaptic plasticity and cellular stress pathways. This spatiotemporal co-localization of metabolism-plasticity-inflammation is highly similar to findings in single-cell studies of Alzheimer’s disease (14), but this study proposes, for the first time, a potential neuronal adaptive response in a psychiatric disorder: it is hypothesized that a state of energetic stress in neurons, potentially linked to altered fatty acid metabolism, might precede or coincide with the activation of inflammatory and apoptotic pathways. However, this proposed model is currently speculative. Direct validation through further *in vitro* experiments or *in vivo* studies is required to test the hypothesized causality between metabolic dysregulation and neuronal inflammation in schizophrenia.

To translate the above findings into clinically applicable information, starting from 158 fatty acid metabolism genes, we employed methods including LASSO regression, univariate logistic regression, and multivariate logistic regression to screen for five core genes—*ACAA1*, *ACAT2*, *ACSS1*, *PSME1*, and *S100A10*—in the training cohort and constructed a multi-gene diagnostic model. The AUC reached 0.856 in the training set and 0.779 in the validation set, significantly outperforming single gene biomarkers. Functionally,*ACAA1* and *ACAT2* are involved in fatty acid oxidation and cholesterol esterification (8, 33), respectively; their dysregulation may impair membrane integrity and synaptic plasticity. *ACSS1* encodes a short-chain fatty acid activation enzyme, and its high expression may promote histone acetylation via excess acetyl-CoA, thereby upregulating pro-inflammatory gene transcription (34), potentially contributing to the hyperactive transcriptional state observed in SCZ; *PSME1*, as an immunoproteasome activator, its downregulation impairs MHC-I-mediated antigen presentation (35); *S100A10* affects excitatory synaptic transmission by regulating calcium channels (36). Therefore, this five-gene signature not only reflects metabolic status but also integrates immune and synaptic function information, providing a concise and robust tool for the early molecular diagnosis of SCZ.

Based on the expression levels of the five genes, we further constructed a nomogram that converts individual risk into a continuous score from 0 to 100. Calibration curves and decision curve analysis showed that the model’s predicted probability highly matched the actual risk and provided significant net benefit within commonly used clinical threshold ranges. Compared to current symptom-based screening tools, this model can provide objective molecular evidence early in the disease course, laying the foundation for moving the intervention window forward; if future non-invasive detection using cfRNA from blood or cerebrospinal fluid becomes feasible, its clinical translation prospects will be even broader.

At the mechanistic level, immune feature score analysis showed that the inflammation level in SCZ neurons was significantly higher than in controls, while the expression of *ACAT2*, *PSME1*, and *ACAA1* was significantly negatively correlated with the chemokine CCL5 and the pro-inflammatory factor IL12B. Based on known biology, *ACAT2* is involved in cholesterol esterification, and its reduced expression could be associated with altered cholesterol homeostasis (37); similarly, *PSME1* plays a role in proteasomal function, and its deficiency might affect inflammatory pathways (38). The observed negative correlations give rise to the hypothesis that reduced expression of these fatty acid metabolism genes might be linked to an amplified neuroinflammatory state, potentially contributing to synaptic and cognitive changes. Most crucially, our single-cell resolution analysis reveals, for the first time within neuronal subpopulations themselves, a strong statistical association between metabolic and immune-related gene expression. This finding shifts the focus beyond traditional microglial studies and suggests a potential interaction between a neuron’s metabolic state and its intrinsic immune response, offering a new perspective for understanding SCZ pathogenesis that warrants functional validation.

Finally, we validated the expression changes of key genes in an MK-801-induced SCZ mouse model: in the DLPFC region, *ACAA1*, *ACAT2*, *PSME1*, and *S100A10* were downregulated (*p* < 0.0001), while *ACSS1* was upregulated (*p* < 0.001), consistent with human data. Notably, these expression patterns were also recapitulated in peripheral blood (**Figure 10**), suggesting that the metabolic dysregulation observed in the brain may be reflected systemically. simultaneously, the model mice exhibited typical hyperactivity and anxiety-like behaviors. Cross-species consistency reinforces the universality of fatty acid metabolism genes participating in SCZ pathology through inflammatory mechanisms, provides strong *in vivo* support for our findings, and offers a basis for subsequent intervention experiments (e.g., *ACSS1* inhibitors or activators of the downregulated genes) as well as the potential development of blood-based diagnostic assays.

Compared to previous studies, this research deeply explores the heterogeneity of neuronal subtypes through single-cell sequencing technology. This cell type-specific perspective complements and extends previous studies based on mixed tissue or plasma. For example, it complements the genome-wide association analysis based on plasma fatty acid concentrations by Jones et al. (39), which revealed a macro-level association between fatty acid metabolism and SCZ but failed to elucidate cell type-specific molecular mechanisms. However, the negative correlation between fatty acid metabolism genes and inflammatory factors (e.g., CCL5, IL12B) found in this study (**Figures 6B–D**) suggests a statistical link between fatty acid metabolism genes and inflammatory factors, raising the possibility that an imbalance in fatty acid metabolism could be associated with neuroinflammatory processes in SCZ. This expands the theoretical framework of metabolic-immune interactions in SCZ pathogenesis, but the directional nature of this relationship (i.e., whether metabolic changes influence inflammation or vice versa) remains unclear. For instance, the observed low expression of *ACAT2* might be part of a broader metabolic state that correlates with inflammatory activity, but the specific mechanisms by which this might occur require direct functional experimental validation.

This study has Several limitations of this study should be acknowledged. The sample size used for model training is relatively modest (9 SCZ patients and 14 controls for single-cell data; GSE174407 as the training cohort), which may affect the statistical power and generalizability of the findings; nevertheless, the use of an independent external validation cohort (GSE107638) helps mitigate this concern by demonstrating consistent model performance across datasets, and future studies incorporating larger, multi-center cohorts and prospective clinical samples are warranted to further validate the robustness and translational potential of this diagnostic model. Beyond the sample size consideration, it is also important to note that this study focused exclusively on the DLPFC region, leaving fatty acid metabolism characteristics in other brain regions implicated in SCZ—such as the hippocampus and striatum—to be elucidated in future work. Moreover, while our diagnostic model was constructed based on transcriptomic data, its clinical applicability would benefit from cross-validation with imaging or behavioral assessments. From a technical perspective, the integration of spatial metabolomics technologies could provide dynamic resolution of metabolite distribution features, thereby deepening our understanding of SCZ pathological mechanisms, and functional validation through approaches such as CRISPR screening or organoid models would help clarify the specific roles and targets of genes like *ACSS1*, offering a foundation for precise diagnostic and therapeutic strategies. Finally, this study did not quantitatively detect metabolites such as ketone bodies; incorporating correlation analyses between metabolite levels and gene expression in future investigations would further elucidate the underlying biological mechanisms and inform precision treatment approaches. Nevertheless, the consistent expression patterns observed in peripheral blood suggest that these transcriptional alterations are not confined to the brain and may have utility as minimally invasive biomarkers, warranting further investigation in larger clinical cohorts.

## 5 Conclusion

This study reveals cell type-specific dysregulation of fatty acid metabolism in SCZ, identifies a robust five-gene diagnostic model validated in both brain and peripheral blood, and provides new insights into the interplay between metabolic and inflammatory pathways in disease pathogenesis. These findings lay a foundation for developing novel diagnostic biomarkers and targeted therapeutic strategies.

## Data Availability

The original contributions presented in the study are included in the article/**Supplementary Material**. Further inquiries can be directed to the corresponding authors.

## Supplementary material

Supplementary Figure 1 Gene Set Variation Analysis (GSVA) revealed differences in signature pathways of various neuronal cell types between SCZ and Control samples.

SUPPLEMENTARY TABLE 1 The complete gene list of the hallmark fatty acid metabolism gene set.

SUPPLEMENTARY TABLE 2 Immune feature gene sets used for single-sample gene set variation analysis (ssGSEA).

SUPPLEMENTARY TABLE 3 Sequences of primers used for quantitative real-time PCR (qPCR).
