# Supplementary figures and images for: Cell type-specific alterations in fatty acid metabolism in neuronal subpopulations of schizophrenia and construction of a diagnostic model
Source: Front Psychiatry. 2026 Apr 21;17:1770038. doi: 10.3389/fpsyt.2026.1770038 (PMC13139169; doi:10.3389/fpsyt.2026.1770038)

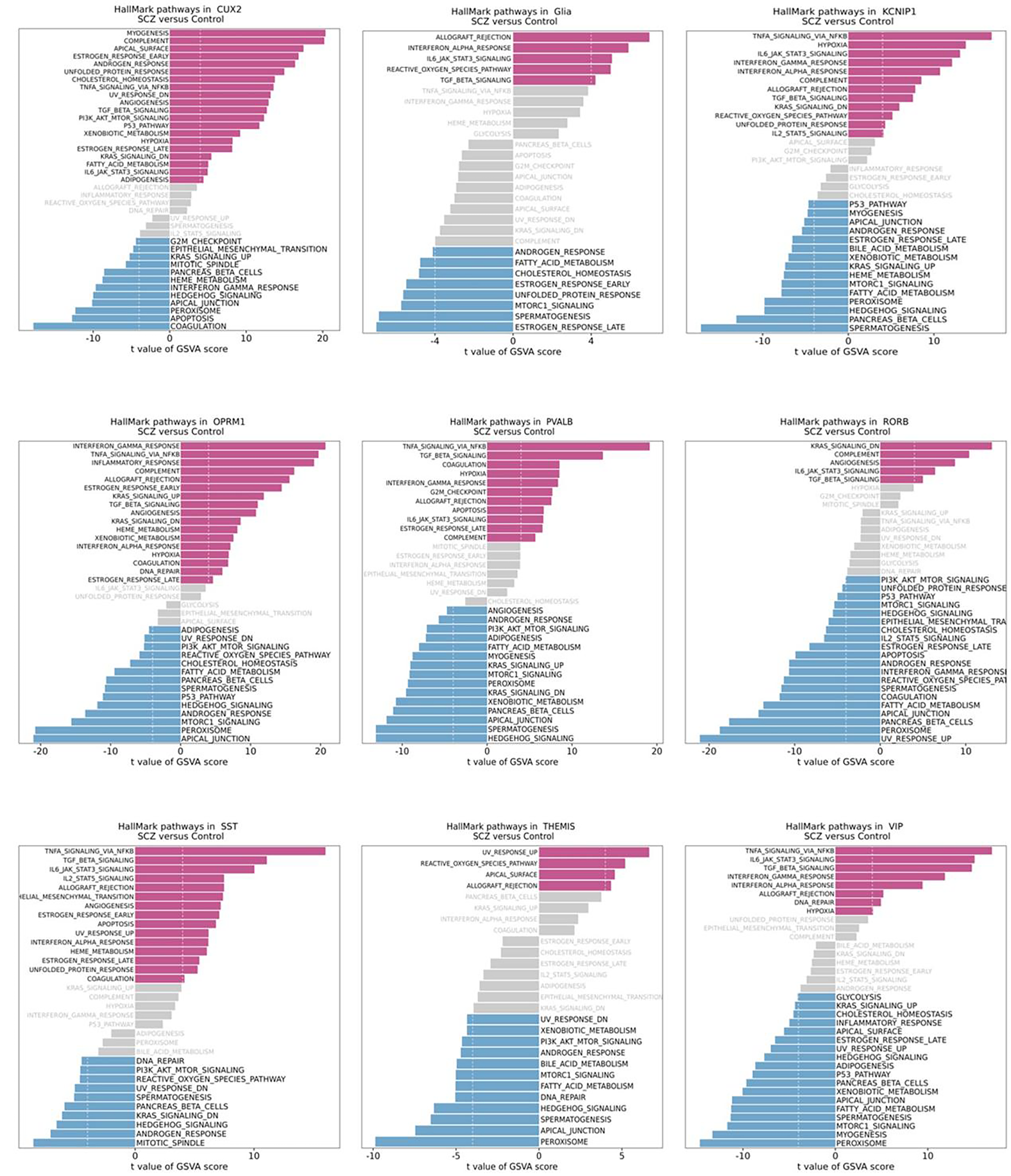

Supplement: Supplementary Figure 1 — Gene Set Variation Analysis (GSVA) revealed differences in signature pathways of various neuronal cell types between SCZ and Control samples. [file Image1.tif]
